# α-Bisabolol alleviates diesel exhaust particle-induced lung injury and mitochondrial dysfunction by regulating inflammatory, oxidative stress, and apoptotic biomarkers through the c-Jun N-terminal kinase signaling pathway

**DOI:** 10.3389/fphar.2024.1485101

**Published:** 2025-01-03

**Authors:** Sumaya Beegam, Nur Elena Zaaba, Ozaz Elzaki, Abderrahim Nemmar

**Affiliations:** Department of Physiology, College of Medicine and Health Sciences, United Arab Emirates University, Al Ain, United Arab Emirates

**Keywords:** diesel exhaust particles, α-bisabolol, airway resistance, oxidative stress, inflammation, mitochondrial dysfunction

## Abstract

**Introduction:**

Exposure to particulate matter ≤2.5 μm in diameter (PM_2.5_) is associated with adverse respiratory outcomes, including alterations to lung morphology and function. These associations were reported even at concentrations lower than the current annual limit of PM_2.5_. Inhalation of PM_2.5_, of which diesel exhaust particles (DEPs) is a major component, induces lung inflammation and oxidative stress. α-Bisabolol (BIS) is a bioactive dietary phytochemical with various pharmacological properties, including anti-inflammatory and antioxidant actions. Here, we evaluated the possible protective effects of BIS on DEP-induced lung injury.

**Methods:**

Mice were exposed to DEPs (20 µg/mouse) or saline (control) by intratracheal instillation. BIS was administered orally at two doses (25 and 50 mg/kg) approximately 1 h before DEP exposure. Twenty-four hours after DEP administration, multiple respiratory endpoints were evaluated.

**Results:**

BIS administration was observed to prevent DEP-induced airway hyperreactivity to methacholine; influx of macrophages, neutrophils, and lymphocytes in the bronchoalveolar lavage fluid; and increases in epithelial and endothelial permeabilities. DEP exposure caused increases in the levels of myeloperoxidase, proinflammatory cytokines, and oxidative stress markers in lung tissue homogenates, and all these effects were abated by BIS treatment. The activities of mitochondrial complexes I, II, III, and IV were markedly increased in the lungs of mice exposed to DEPs, and these effects were significantly reduced in the BIS-treated group. Intratracheal instillation of DEPs induced DNA damage and increase in the apoptotic marker cleaved caspase-3. The latter effects were prevented in mice treated with BIS and exposed to DEPs. Moreover, BIS mitigated DEP-induced increase in the expression of phospho-c-Jun N-terminal kinase (JNK) in a dose-dependent manner.

**Discussion:**

BIS markedly alleviated DEP-induced lung injury by regulating the inflammatory, oxidative stress, and apoptotic biomarkers through the JNK signaling pathway. Following additional studies, BIS may be considered as a plausible protective agent against inhaled-particle-induced pulmonary adverse effects.

## Introduction

Multiple epidemiological studies have shown that short-term elevations in the concentrations of particulate air pollutants with aerodynamic diameters ≤2.5 μm (PM_2.5_) are associated with increased prevalence of respiratory symptoms and pulmonary function impairments, including decreased forced vital capacity (FVC), forced expiratory volume in one second (FEV1), and ratio FEV1/FVC (Xing et al., 2016; Dominski et al., 2021; Hachem et al., 2021; Peden, 2024). Exhaust from diesel engines is a significant contributor to PM_2.5_ in urban areas, in addition to being a main source of combustion-derived nanoparticles (Miller and Newby, 2020; Portugal et al., 2024). These particles can profoundly enter the respiratory tract and have large surface areas on which they can transport significant quantities of noxious compounds, including hydrocarbons and metals (Miller and Newby, 2020; Portugal et al., 2024).

Inhaled particles can trigger toxicity through various mechanisms, such as activation of the sensory receptors of the autonomic nervous system, pulmonary and systemic inflammatory reactions, and air-to-blood-barrier translocation (Nemmar et al., 2013; Steiner et al., 2016; Henning, 2024). Evidence suggests that inhaled diesel exhaust particles (DEPs) precipitate these effects via the oxidative stress pathways (Nemmar et al., 2013; Steiner et al., 2016; Henning, 2024). Controlled exposure investigations in humans have shown robust associations between inhaled particulate air pollutants and markers of pulmonary oxidative stress in healthy people and persons with established lung diseases (e.g., chronic obstructive pulmonary diseases and asthma) (Laumbach et al., 2014; Xu et al., 2013). Similarly, various experimental animal studies have reported that pulmonary exposure to DEPs can induce increased airway resistance, lung inflammation, and oxidative stress (Nemmar et al., 2018; Zin et al., 2012; Hartz et al., 2008).

α-Bisabolol (BIS) is a sesquiterpene alcohol present in essential oils originating from several plants (e.g., *Matricaria recutita*, Lamiaceae, Myrtaceae, and Apiaceae) (Eddin et al., 2022). BIS is a widely used ingredient in cosmetic and dermatological formulations and has been proven to be safe when administered orally to rodents (median lethal dose: 13–14 g/kg bodyweight) (Eddin et al., 2022). Notably, BIS has been reported to have antioxidant, anti-inflammatory, anticancer, cardioprotective, gastroprotective, and nephroprotective properties (Eddin et al., 2022; Zaaba et al., 2022; Rocha et al., 2011; Meeran et al., 2020; Mf et al., 2024). However, the effects of BIS on DEP-induced pulmonary toxicity have not been studied before. It is well-established that pulmonary adverse effects of particulate air pollution persist even at concentrations lower than the current annual limit of PM_2.5_ (Christidis et al., 2019; Strak et al., 2021); therefore, it is important to adopt additional measures aimed at preventing or alleviating the pathophysiological effects of inhaled particulate air pollutants through the consumption of safe dietary supplements (Miller and Newby, 2020; Guo et al., 2024).

Thus, the aim of the present work is to evaluate the possible protective effects and underlying action mechanisms of BIS on pulmonary toxicity induced by DEPs through the assessment of various pulmonary endpoints, including airway hyperresponsiveness, inflammation, oxidative stress, mitochondrial function, DNA damage, apoptosis, and phospho-c-Jun N-terminal kinase (JNK) expression.

## Materials and methods

### Animals and treatments

This project was appraised and accepted by the Institutional Review Board of the United Arab Emirates University (UAEU), and all experiments were conducted as per the protocol approved by the UAEU Animal Research Ethics Advisory Committee (ERA_2021_8443, approved on 02-01-2022).

### DEPs and mouse treatments

The DEPs used in the present work were acquired from the National Institute of Standards and Technology (Gaithersburg, MD, United States). They were suspended in sterile sodium chloride (0.9%) containing Tween 80 (0.01%). To abate their aggregation, the DEP suspensions were sonicated using an ultrasonic bath (Clifton, NJ, United States) for 15 min and vortexed prior to dilution and intratracheal (i.t.) instillation. The control mice were i.t. instilled with saline containing Tween 80 (0.01%). The DEPs were previously examined via transmission electron microscopy and found to include considerable amounts of nanosized and larger particle aggregates (Nemmar et al., 2007).

BALB/C mice (from the animal house of College of Medicine and Health Sciences, UAEU) of both genders weighing 20–25 g and aged 6–8 weeks were housed in lighting (12/12 h light/dark cycle) and temperature (22°C ± 1°C) controlled rooms. They were allowed unrestricted access to commercially available laboratory chow and provided with tap water on an *ad libitum* basis. The total number of mice used in this study was 126. The numbers of male and female mice used to evaluate the different endpoints were similar among the studied groups. To assess the airway hyperreactivity to methacholine experiments, a separate set of animals was used (n = 8 × 6 groups = 48 mice, with each group including 4 male and 4 female mice). For the DNA damage assessment, as the analysis had to be performed on freshly collected samples, we used a separate set of mice (n = 5 × 6 groups = 30 mice, with 3 males and 2 females in each group). To evaluate all the other biochemical parameters, we used n = 8 × 6 groups = 48 mice (with each group including 4 males and 4 females).

In this study, we assessed the acute (24 h) impacts of DEPs on lung toxicity and the possible protective effects of BIS thereof. Lung deposition of the DEPs was accomplished by i.t. instillation (Nemmar et al., 2018, 2011a). The mice were anesthetized with isoflurane and positioned supine with their necks extended on an angled board. A 24-gauge cannula (Becton Dickinson, Franklin Lakes, NJ, United States) was introduced through the mouth into the trachea. The DEP suspension (20 μg/mouse) or vehicle was i.t. instilled (100 µL) via a sterile syringe and followed by 100 µL of air bolus. BIS purchased from Sigma Aldrich Co. (St. Louis, MO, United States) was diluted in sunflower oil and administered by gavage in two doses of 25 and 50 mg/kg approximately 1 h prior to exposure to the DEPs (Zaaba et al., 2022; Javed et al., 2020). This dosage of BIS is comparable to the regimens applied by other researchers for other conditions (Zaaba et al., 2022; Meeran et al., 2020; Heimfarth et al., 2022). The mice were then randomly separated into six equal groups for the following treatments:

• Group 1 (n = 8): control mice received sunflower oil (10 mL/kg) administered by gavage 1 h prior to i.t. instillation of saline.
• Group 2 (n = 8): mice received BIS at 25 mg/kg dissolved in sunflower oil (10 mL/kg) and administered by gavage 1 h prior to i.t. instillation of saline.
• Group 3 (n = 8): mice received BIS at 50 mg/kg dissolved in sunflower oil (10 mL/kg) and administered by gavage 1 h prior to i.t. instillation of saline.
• Group 4 (n = 8): mice received sunflower oil (10 mL/kg) administered by gavage 1 h prior to i.t. instillation of DEPs (20 μg/mouse).
• Group 5 (n = 8): mice received BIS at 25 mg/kg dissolved in sunflower oil (10 mL/kg) and administered by gavage 1 h prior to i.t. instillation of DEPs (20 μg/mouse).
• Group 6 (n = 8): mice received BIS at 50 mg/kg dissolved in sunflower oil (10 mL/kg) and administered by gavage 1 h prior to i.t. instillation of DEPs (20 μg/mouse).

Twenty-four hours after i.t. instillation of either DEPs or saline, multiple physiological, biochemical, and molecular parameters were evaluated.

### Airway hyperreactivity responses to methacholine

In a separate set of animals (n = 8 per group), the airway hyperreactivity responses were assessed via the forced oscillation technique (FlexiVent, SCIREQ, Montreal, Canada) (Nemmar et al., 2018, 2021). The airway resistance (R) was evaluated following supplementation of increasing concentrations of methacholine (0–40 mg/mL). The animals were anesthetized with 70 mg/kg pentobarbital administered via intraperitoneal injection. The trachea was then exposed to introduce a metal needle (18 gauge). The mice were placed in a computer-monitored small-animal ventilator and quasi-sinusoidally ventilated at a tidal volume of 10 mL/kg, respiratory rate of 150 breaths/min, and positive end-expiratory pressure of 2 cm H_2_O to achieve a mean pulmonary volume close to that observed in spontaneous respiration. After baseline assessment, each animal was challenged with methacholine aerosol produced using an in-line nebulizer and supplied directly via the ventilator for 5 s in augmenting concentrations (0, 0.625, 2.5, 10, and 40 mg/mL). The value of R was quantified through a “snapshot” protocol of 20 s each for 2 min. The mean of these five values was used for each methacholine concentration except in instances where the coefficient of determination of a measurement was less than 0.95. For each animal, R was plotted against the methacholine concentration (from 0 to 40 mg/mL) (Nemmar et al., 2018, 2021).

### Bronchoalveolar fluid (BALF) collection and analysis

The BALF was harvested and examined in accordance with a previously reported technique (Nemmar et al., 2018, 2021, 2011b). In brief, the animals were euthanized with an overdose of sodium pentobarbital after exposure to either saline or DEPs with and without BIS treatment. The trachea was cannulated and the lungs were lavaged thrice with 0.7 mL (total volume of 2.1 mL) of a sterile solution of NaCl (0.9%). The collected BALF samples were pooled, and there was no variation in the volumes of collected BALFs between various groups. The BALF was centrifuged at 1,000*g* for 10 min at 4°C, and the supernatant was stored at −80°C until further analysis for the total proteins (using a kit from Bio-Rad, Munich, Germany). The pellets were resuspended and stained with 1% gentian violet, following which the cells were counted using a Thoma hemocytometer. The cell differentials were accomplished microscopically on cytocentrifuge preparations that were first fixed in methanol and then stained with Diff-Quick (Dade Behring AG, Düdingen, Switzerland).

### Evans Blue extravasation

To evaluate vascular leakage, the mice exposed to either saline or DEPs with and without BIS treatment were injected with Evans Blue dye (20 mg/kg) via the tail vein 30 min before the end of the experiment (Jacob et al., 2009). Following pulmonary perfusion with phosphate-buffered saline (PBS), the lungs were removed and dried on filter paper before being weighed and snap frozen in liquid nitrogen for storage at −80°C. On the day of the analysis, the lung tissues were homogenized and incubated with two volumes of formamide (Fisher Scientific, Fair Lawn, NJ, United States) for 18 h at 60°C. The supernatants were then harvested after centrifugation at 5,000*g* for 30 min. The optical density of the supernatant was measured via spectrophotometry at 620 nm. The concentrations of the Evans Blue dye in the collected samples were assessed through a standard curve prepared with known concentrations of the dye, and the data were expressed in terms of micrograms per gram of the lung (Jacob et al., 2009).

### Lung wet to dry weights ratio

The ratio of lung wet to dry weights was used as the index of lung edema development and reflected the increase in pulmonary permeability (Jacob et al., 2009; Nemmar et al., 2008). The lung tissues were weighed immediately after collection to obtain the wet weight (WW) and then placed in an oven at 70°C for 48 h. Thereafter, the lungs were weighed again to obtain the dry weight (DW), following which WW/DW was calculated.

### Measurement of inflammation and oxidative stress markers in lung homogenates

Following i.t. instillation of either saline or DEPs with and without BIS treatment, the mice were euthanized by an overdose of sodium pentobarbital, and their lungs were promptly harvested and rinsed with ice-cold PBS (pH 7.4) prior to homogenization, as reported in a previous work (Nemmar et al., 2018, 2015). The homogenates were centrifuged at 3,000*g* for 10 min to eliminate cellular debris, and the supernatants were used for additional analyses (Nemmar et al., 2018, 2015). The protein concentrations was measured using Bradford’s method.

The concentrations of myeloperoxidase (MPO), tumor necrosis factor α (TNFα), and interleukins (ILs) 6 and 1β were determined using ELISA kits purchased from R&D Systems (Minneapolis, MN, United States) (Al Za’abi et al., 2018; Ali et al., 2019). NADPH-dependent membrane lipid peroxidation (LPO) was quantified as the thiobarbituric acid reactive substance (TBARS) using malondialdehyde as the standard (Sigma-Aldrich Fine Chemicals, St. Louis, MO, United States). The glutathione (GSH) concentrations were measured following manufacturer protocol provided with a commercially available kit from Sigma-Aldrich Fine Chemicals (Munich, Germany). The superoxide dismutase (SOD) activity was determined spectrophotometrically using a commercially available kit from Cayman Chemical (Ann Arbor, MI, United States). Nitric oxide (NO) levels were quantified using a total NO assay based on a commercially available kit from R&D Systems (Minneapolis, MN, United States) that measures stable nitrites (NO₂⁻) and nitrates (NO₃⁻) (Al Za’abi et al., 2018; Ali et al., 2019).

### Mitochondrial respiratory complex activities in the lung

The enzyme activities of the mitochondrial respiratory complexes I, II, III, and IV were measured using previously described techniques (Wieckowski et al., 2009; Agarwal et al., 2012).

### DNA damage in the lung

DNA damage in the lungs of mice exposed to either saline or DEPs with and without BIS pretreatment were evaluated using the Comet assay, as reported earlier (Hartmann and Speit, 1997; Nemmar et al., 2016). The estimation of DNA migration encompassing the nuclear diameter and migrated DNA was achieved using image analysis with Axiovision 3.1 software (Carl Zeiss, Toronto, ON, Canada), as described in earlier works (Hartmann and Speit, 1997; Nemmar et al., 2016).

### Cleaved caspase-3 in the lung

The cleaved caspase-3 levels in the lung tissues of the control and DEP-exposed mice with and without BIS treatments were quantified using ELISA kits from R&D Systems (Minneapolis, MN, United States).

### Western blot analysis for quantifying JNK expression

The protein expressions of JNK were measured from the lung tissues harvested from mice that were i.t. instilled with either saline or DEPs with and without BIS administration through Western blotting, as described before (Suchal et al., 2016).

## Statistics

All statistical analyses were performed using GraphPad Prism Software version 7. Comparisons between the groups were performed by two-way analysis of variance (ANOVA), followed by Holm–Sidak’s multiple comparisons test. All the data in figures were reported as mean ± standard error of the mean (SEM), and *p* values <0.05 were considered to be significant.

## Results

### Airway hyperresponsiveness to methacholine

**FIGURE 1:**
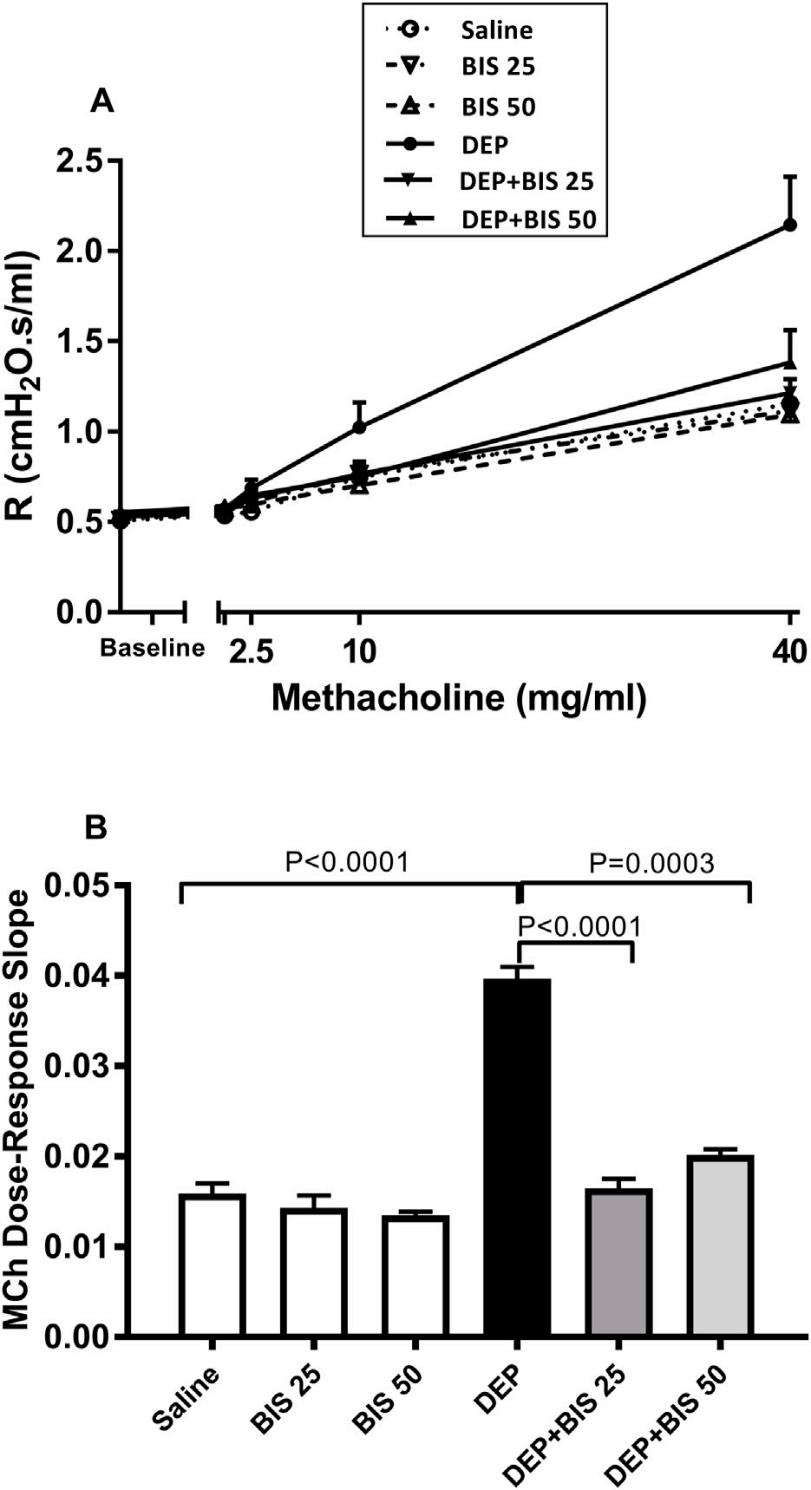
Airway hyperresponsiveness based on the airway resistance (*R*), following exposure to increasing methacholine (MCh) concentrations (0–40 mg/mL), was assessed via the forced oscillation technique 24 h after pulmonary exposure to either saline or diesel exhaust particles (DEPs, 20 µg/mouse) with and without bisabolol (BIS) treatment. **(A)** Total dose–response curves of the respiratory system resistance to increasing doses of MCh. **(B)** From the resistance MCh dose–response curves in **(A)**, the index of airway hyperresponsiveness was calculated as the slope of the linear regression using 0–40 mg/mL of MCh concentrations. The data are shown as mean ± SEM (*n* = 8 per group).

The forced oscillations technique was used to evaluate the airway resistance in mice following exposure to increasing methacholine concentrations after i.t. instillation with either saline or DEPs with and without BIS treatment. Compared to the control group, there was a dose-dependent increase in airway resistance in the DEP-exposed group. No differences were observed in the saline vs. BIS 25 mg/kg or BIS 50 mg/kg groups. Remarkably, treatments with both doses of BIS abated DEP-induced airway hyperresponsiveness following exposure to increasing concentrations of methacholine (Figure 1A). As seen in Figure 1A, the index of airway responsiveness was estimated from the slope of the linear regression curve of methacholine concentrations used (Figure 1B), which showed a significant increase in the i.t. DEP instilled group compared to the control group (*p* <0.0001) as well as significant protective effects for both doses of BIS against DEPs (*p* <0.0001 to P = 0.0003) (Figure 1B).

### BALF cellular composition

**FIGURE 2:**
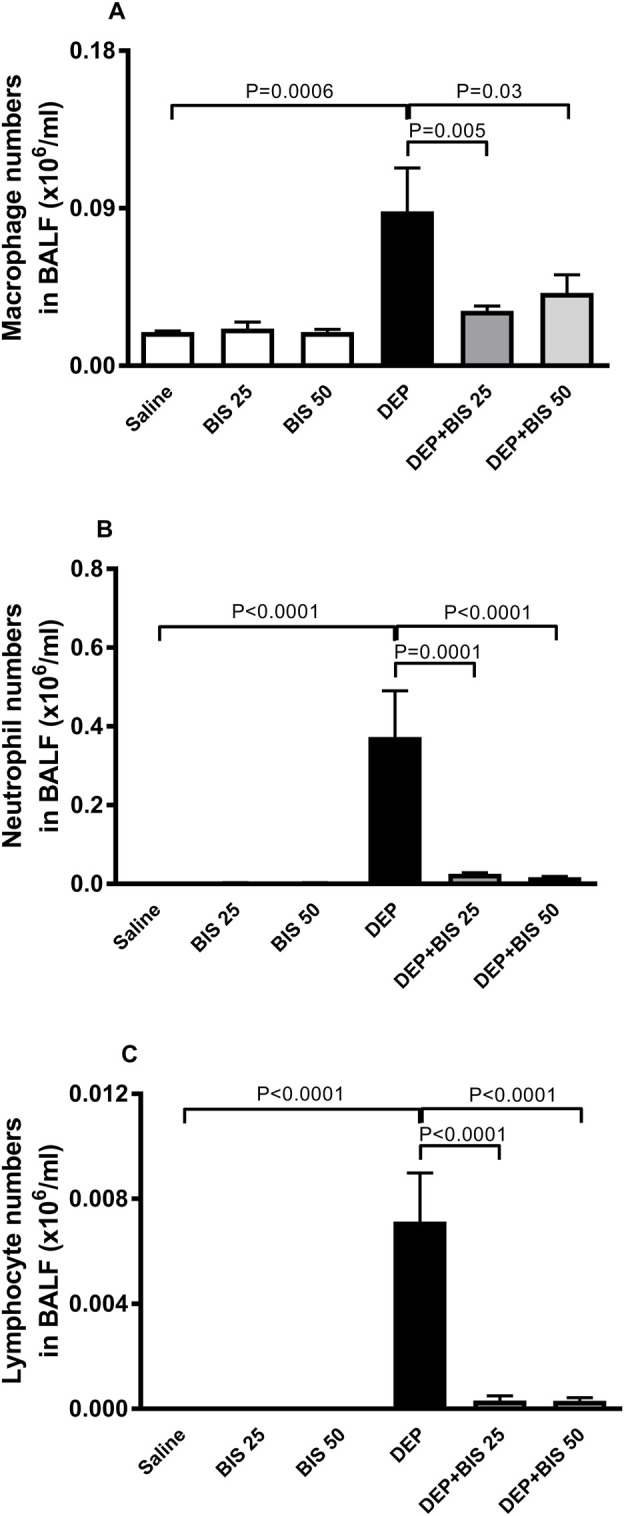
Numbers of **(A)** macrophages, **(B)** polymorphonuclear neutrophils and **(C)** lymphocytes in the bronchoalveolar lavage fluid (BALF) 24 h after pulmonary exposure to either saline or diesel exhaust particles (DEPs, 20 µg/mouse) with and without bisabolol (BIS) treatment. The data are shown as mean ± SEM (n = 8 per group).

Figure 2 shows that compared with the control group, i.t. instillation of DEPs induced significant increases in the numbers of macrophages (Figure 2A), neutrophils (Figure 2B), and lymphocytes (Figure 2C) (*p* <0.0001 to P = 0.0006). These effects were significantly prevented in mice that were concomitantly administered DEPs along with BIS 25 mg/kg or 50 mg/kg (*p* <0.0001 to P = 0.03).

### WW/DW ratio, Evans Blue extravasation, and BALF protein content

**FIGURE 3:**
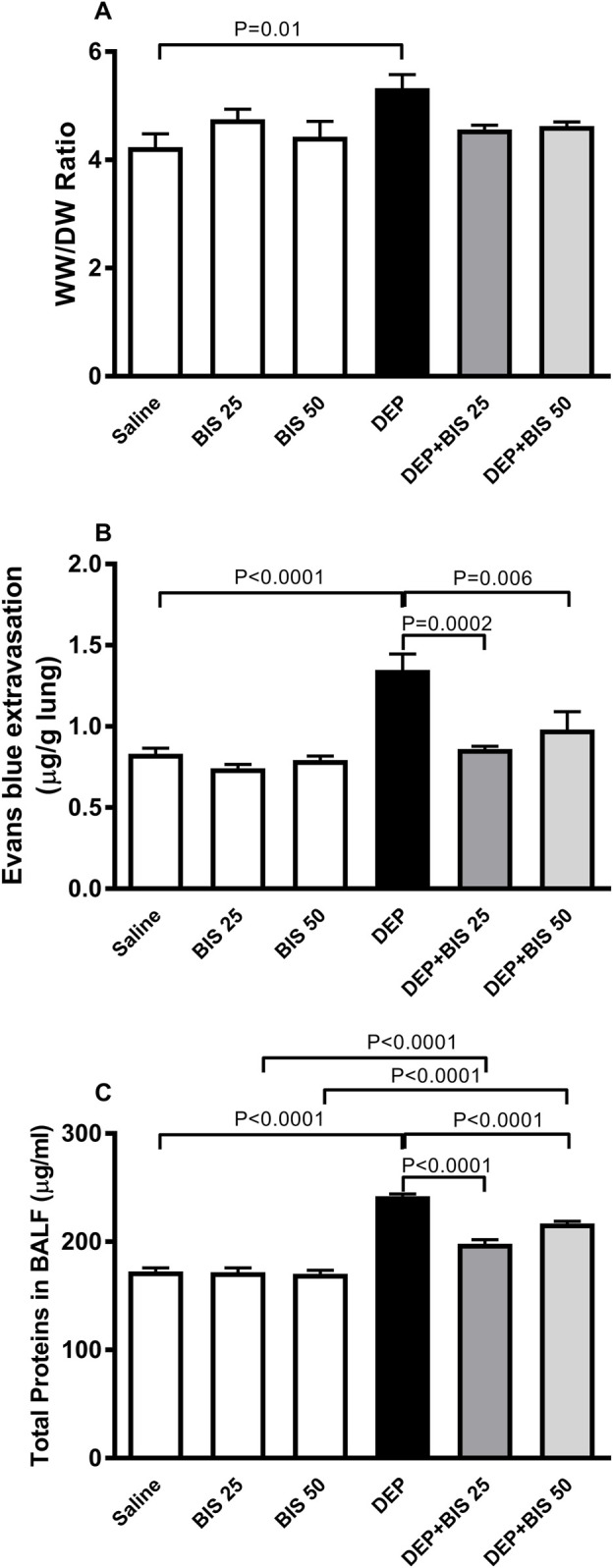
**(A)** Wet to dry weights ratio (WW/DW), **(B)** Evans Blue extravasation, and **(C)** total proteins in the bronchoalveolar lavage fluid (BALF) 24 h after pulmonary exposure to either saline or diesel exhaust particles (DEPs, 20 µg/mouse) with and without bisabolol (BIS) treatment. The data are shown as mean ± SEM (n = 8 per group).

The lungs of mice exposed to DEPs showed significantly higher WW/DW ratio (*p* = 0.01), Evans Blue extravasation (*p* <0.0001), and total protein content in the BALF (*p* <0.0001) compared to animals in the saline-exposed group (Figures 3A–C). The combination of DEPs with BIS 25 mg/kg or 50 mg/kg significantly mitigated augmentation in Evans Blue extravasation (*p* = 0.0002–0.006) and the total protein content in the BALF (*p* <0.0001). The administration of DEPs with BIS 25 mg/kg or 50 mg/kg prevented increase in the WW/DW ratio induced by DEPs; however, this effect did not reach statistical significance.

### MPO activity and TNFα, IL-1β, and IL-6 concentrations in lung homogenates

**FIGURE 4:**
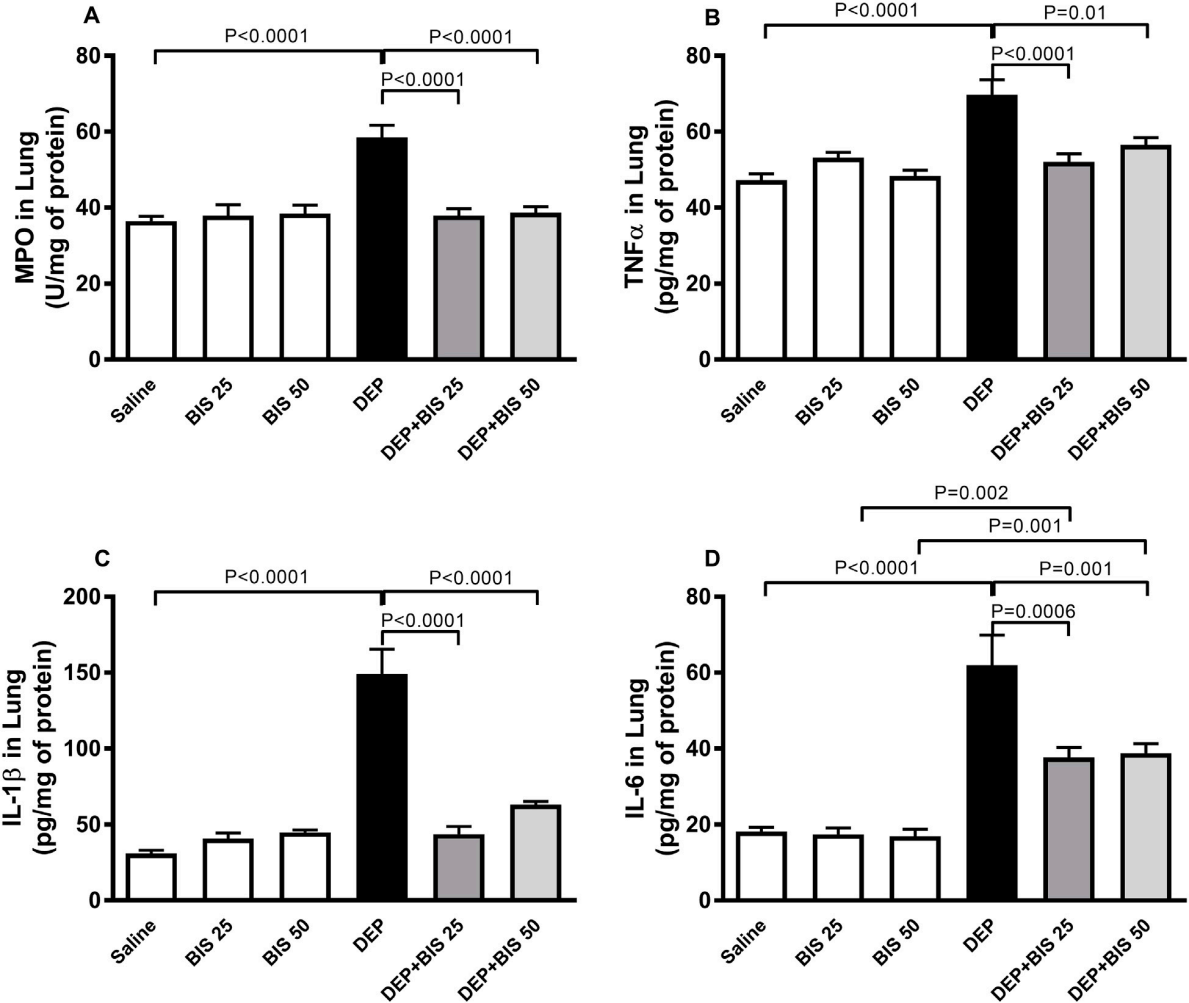
**(A)** Myeloperoxidase (MPO) activity as well as concentrations of **(B)** tumor necrosis factor α (TNFα), **(C)** interleukin 1β (IL-1β), and **(D)** interleukin 6 (IL-6) in the lung tissues 24 h after pulmonary exposure to either saline or diesel exhaust particles (DEPs, 20 µg/mouse) with and without bisabolol (BIS) treatment. The data are shown as mean ± SEM (n = 8 per group).

Compared to the control group, pulmonary exposure to DEPs significantly augmented MPO (*p* <0.0001; Figure 4A) activity along with TNFα (*p* <0.0001; Figure 4B), IL-1β (*p* <0.0001; Figure 4C), and IL-6 (*p* <0.0001; Figure 4D) concentrations in the lung homogenates. Following administration of DEPs with BIS 25 mg/kg or 50 mg/kg, there were significant reductions in the increase of MPO, TNF-α, IL-1β, and IL-6 levels in the lung homogenates (*p* <0.0001 to P = 0.01).

### LPO, SOD, GSH, and NO levels in the lung homogenates

**FIGURE 5:**
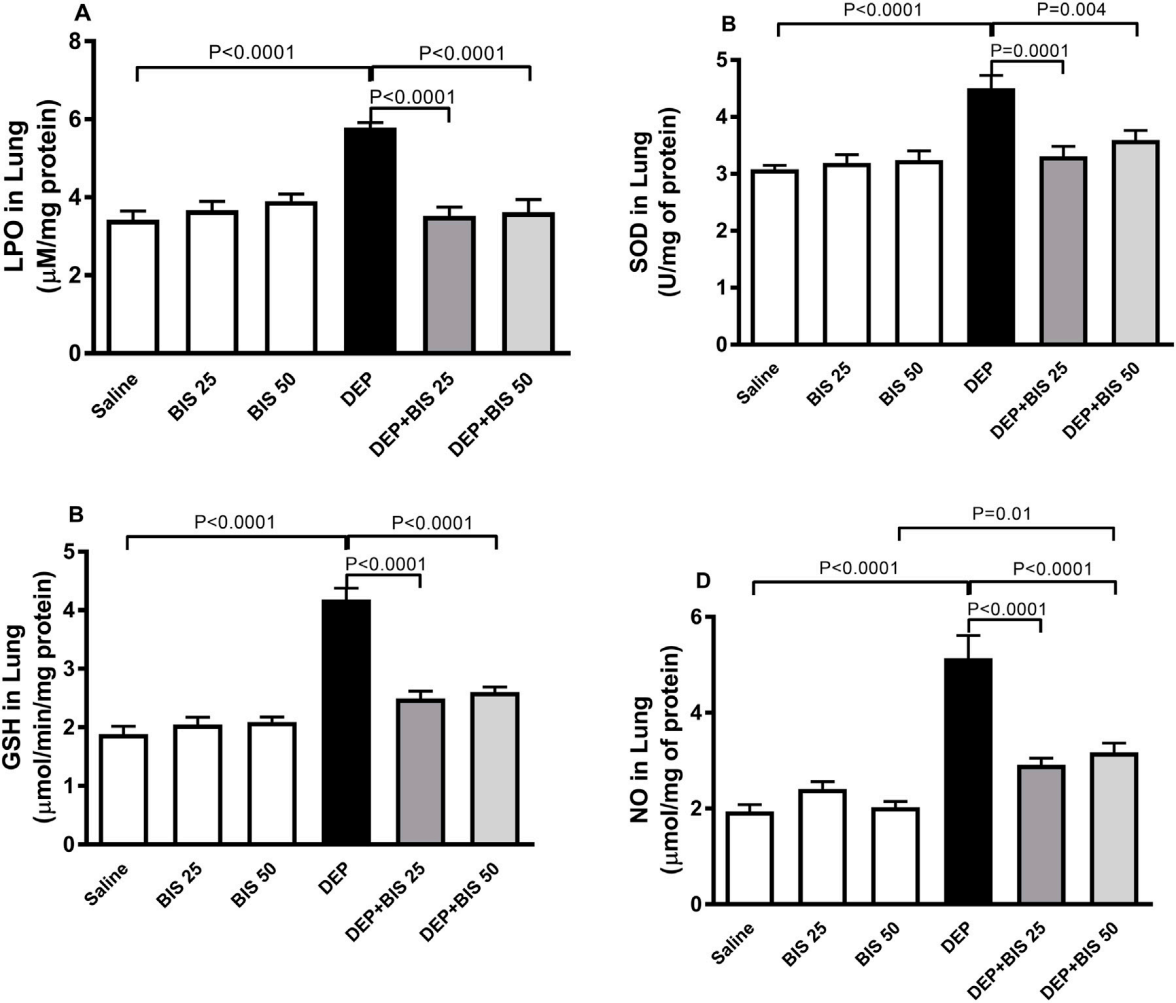
**(A)** Lipid peroxidation (LPO), **(B)** superoxide dismutase (SOD), **(C)** glutathione (GSH), and **(D)** total nitric oxide (NO) levels in the lung tissues 24 h after pulmonary exposure to either saline or diesel exhaust particles (DEPs, 20 µg/mouse) with and without bisabolol (BIS) treatment. The data are shown as mean ± SEM (n = 8 per group).

Figure 5 illustrates the effects of DEP alone, DEP + BIS 25 mg/kg, and DEP + BIS 50 mg/kg on the oxidative and nitrosative stress markers. The levels of LPO, SOD, GSH, and NO were markedly higher following pulmonary exposure to DEPs (*p* <0.0001). Compared to the DEP group, concomitant administration DEP with BIS 25 mg/kg or 50 mg/kg prevented increases in the levels of LPO (*p* <0.0001), SOD (*p* = 0.0001–0.004), GSH (*p* <0.0001), and NO (*p* <0.0001).

### Mitochondrial respiratory complex activities in the lung

**FIGURE 6:**
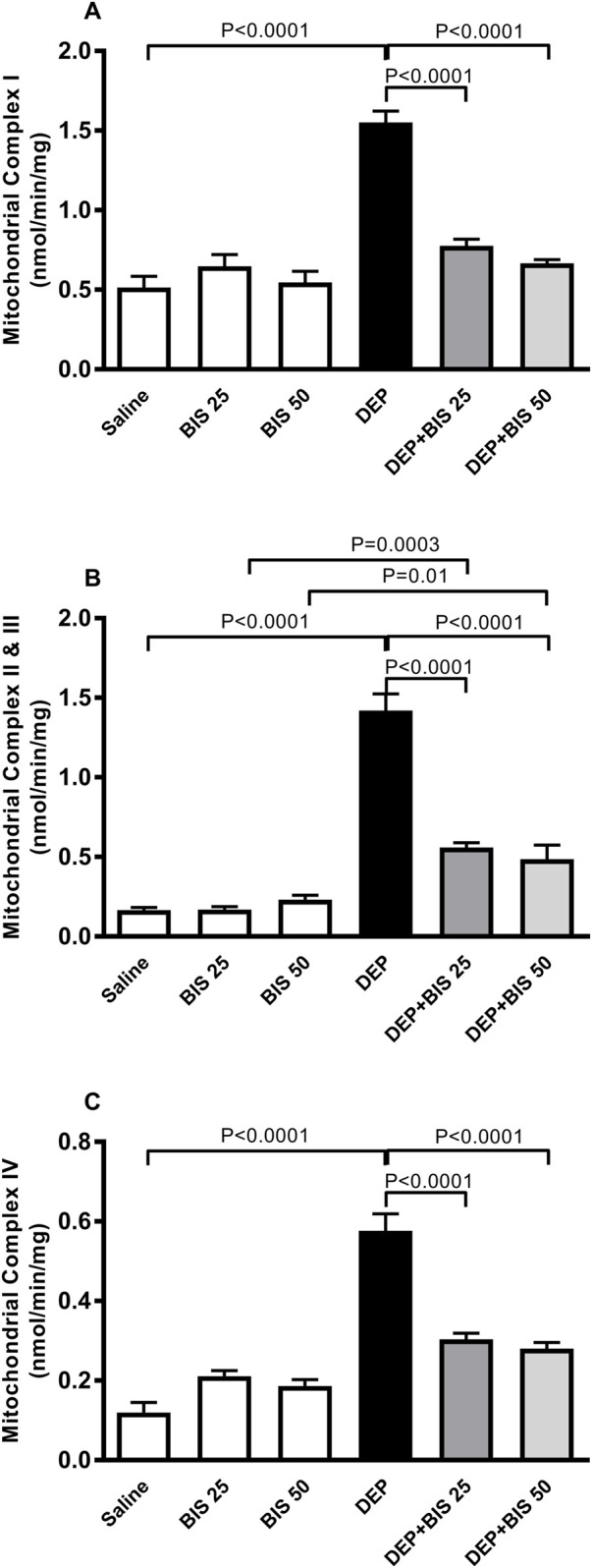
Activities of the mitochondrial respiratory complexes **(A)** I, **(B)** II and III, and **(C)** IV in the lung tissues 24 h after pulmonary exposure to either saline or diesel exhaust particles (DEPs, 20 µg/mouse) with and without bisabolol (BIS) treatment. The data are shown as mean ± SEM (n = 8 per group).

Figure 6A shows that compared to saline exposure, i.t. instillation with DEPs induced a remarkable elevation in the activity of the mitochondrial respiratory complex I (*p* <0.0001). This effect was dose-dependently abated when either BIS 25 mg/kg or 50 mg/kg were coadministered with DEPs compared to the group of mice instilled DEPs alone (*p* <0.0001). Similarly, as shown in Figure 6B, the activities of the mitochondrial respiratory complexes II and III were significantly augmented in the DEP-exposed group than the saline-exposed animals (*p* <0.0001). The effects of the latter were alleviated in a dose-dependent manner in the DEP + BIS 25 mg/kg (*p* <0.0001) and DEP + BIS 50 mg/kg (*p* <0.0001) animals compared to the DEP group. As shown in Figure 6C, pulmonary exposure to DEPs prompted significantly elevated activity of the mitochondrial respiratory complex IV in the lung (*p* <0.0001), and administration of either BIS 25 mg/kg (*p* <0.0001) or 50 mg/kg (*p* <0.0001) 1 h before exposure to the DEPs markedly mitigated these effects.

### DNA migration and cleaved caspase-3 in the lung

**FIGURE 7:**
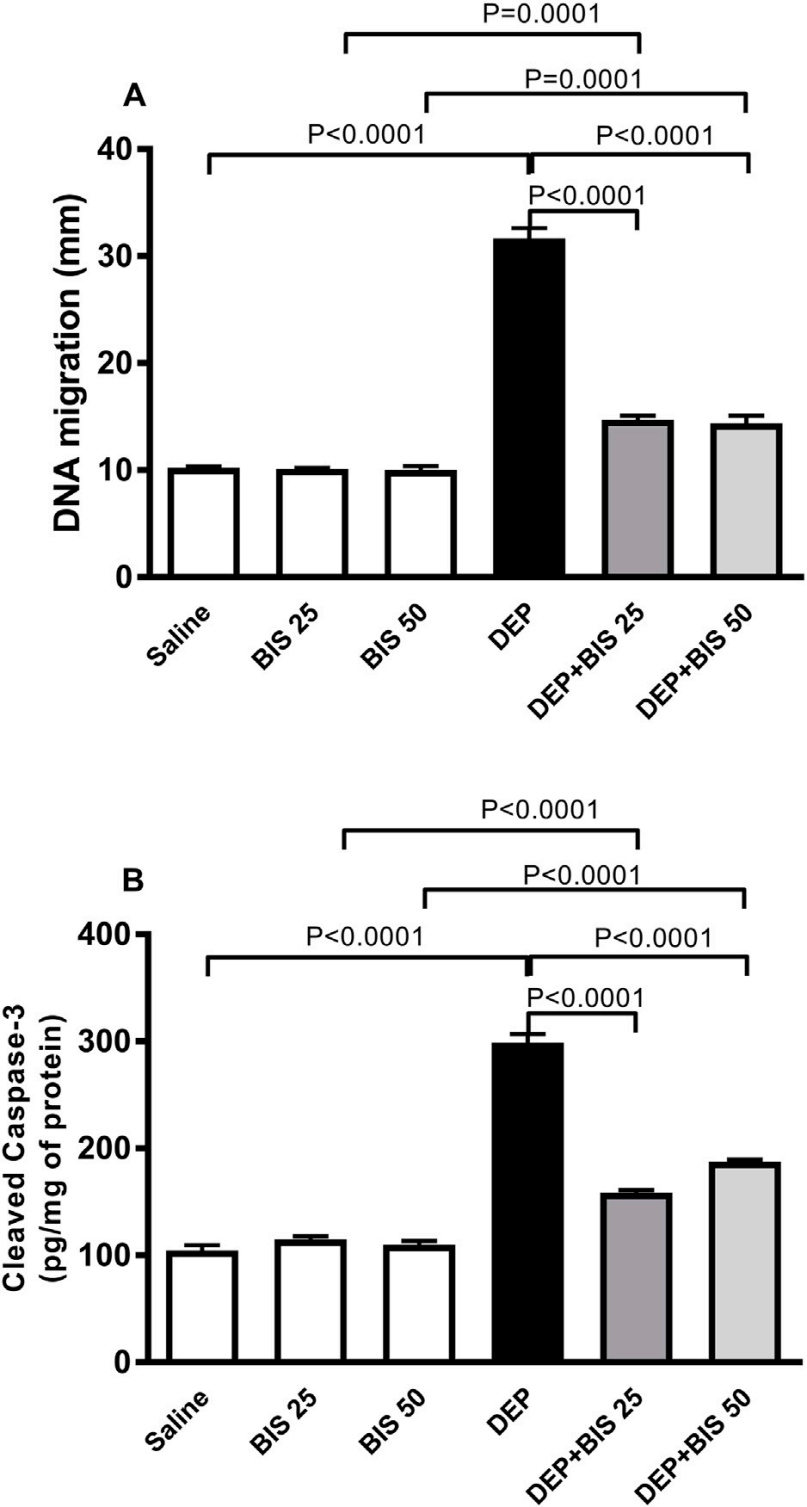
**(A)** DNA damage assessed by the Comet assay and **(B)** cleaved caspase-3 concentrations in the lung tissues 24 h after pulmonary exposure to either saline or diesel exhaust particles (DEPs, 20 µg/mouse) with and without bisabolol (BIS) treatment. The data are shown as mean ± SEM (n = 5 per group for DNA damage; n = 8 for cleaved caspase-3 concentration).

Figure 7 shows the impacts of i.t. instillation of DEPs on DNA damage and concentration of cleaved caspase-3 in the lung tissue homogenate as well as the protective effects of BIS. Exposure to DEPs significantly elevated both DNA migration signifying DNA damage (*p* <0.0001) and concentration of cleaved caspase-3 (*p* <0.0001). Compared to the DEP-exposed group, concomitant administration of DEPs with BIS 25 mg/kg or 50 mg/kg significantly ameliorated DNA damage (*p* <0.0001; Figure 7A) and apoptosis (*p* <0.0001; Figure 7B).

### JNK levels in the lung tissue homogenate

**FIGURE 8:**
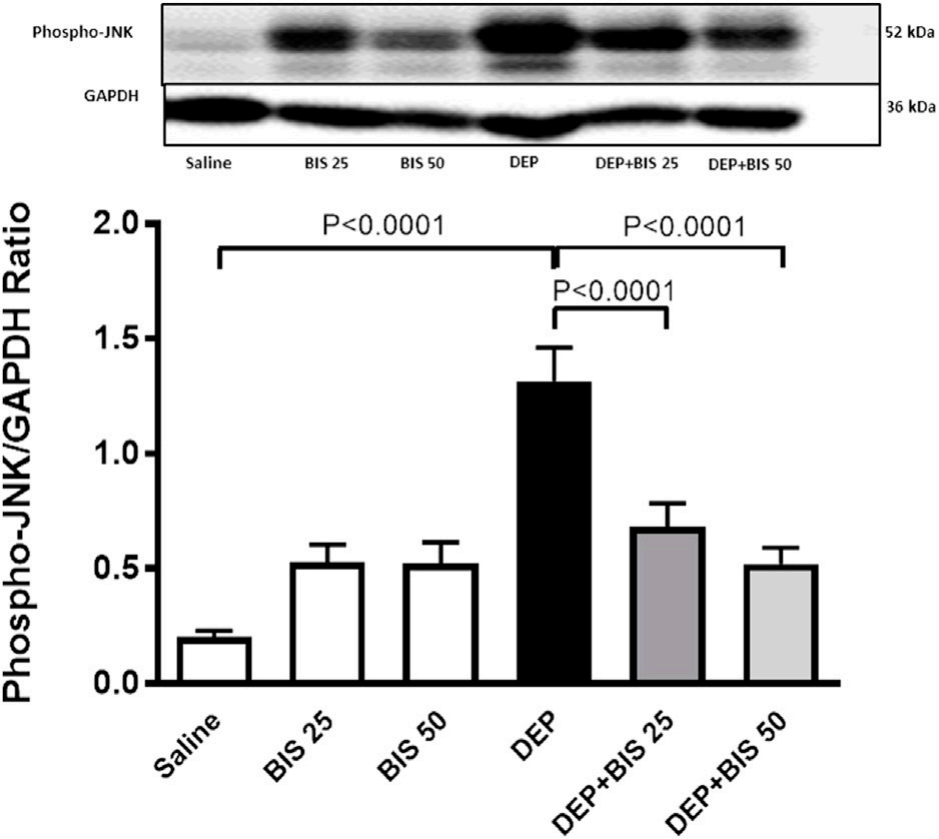
Expression of phosphorylated c-Jun N-terminal kinase (JNK) in the lung tissue assessed via Western blotting 24 h after pulmonary exposure to either saline or diesel exhaust particles (DEPs, 20 µg/mouse) with and without bisabolol (BIS) treatment. The data are shown as mean ± SEM (n = 8 per group).

Figure 8 shows that compared to saline, DEP exposure caused significant augmentation in the levels of JNK in the lung tissue homogenate (*p* <0.0001). There were slight and statistically insignificant increases in the JNK concentrations in the BIS 25 mg/kg and 50 mg/kg vs. saline groups. Interestingly, in the mice given DEP with BIS 25 mg/kg (*p* <0.0001) or 50 mg/kg (*p* <0.0001), the concentrations of JNK decreased in a dose-dependent manner.

## Discussion

In this study, we provide evidence that BIS alleviates DEP-induced lung injury by regulating inflammation, oxidative stress, mitochondrial dysfunction, DNA damage, and apoptosis through the JNK signaling pathway. The global mortality burden is significantly attributable to particulate air pollution; in particular, PM_2.5_ has been reported to cause over 4 million deaths yearly, which represents about 7.6% of all deaths from all causes (Cohen et al., 2017). Furthermore, it has been demonstrated that short-term exposure to particulate air pollutants is accompanied by increased risks for all causes of mortality, in addition to risk from asthma and chronic obstructive pulmonary disease (Liu et al., 2022; Liu et al., 2019; Li et al., 2017; Gouveia et al., 2024; Yu et al., 2024). It has also been reported that short-term exposure to particulate matter is associated with elevated risks of respiratory symptoms, respiratory disease exacerbation, emergency department visits, and hospital admission (Yu et al., 2024; Fajersztajn et al., 2017).

A recent study conducted on the total populations of seven US states with more than a million mortalities revealed that short-term exposure to particulate air pollution is independently linked with elevated risk to all-cause mortality; importantly, it was also shown that the impacts of air pollution were persevered even at low PM_2.5_ concentrations (Liu et al., 2022). Hence, along with reevaluation of the existing daily standards of PM_2.5_ levels, it is important to adopt additional interventions to reduce the pathophysiological effects of air pollution, including the use of safe phytochemicals with antioxidant and anti-inflammatory properties (Miller and Newby, 2020; Guo et al., 2024).

In the present study, we assessed the acute effects of i.t. instilled DEPs and the protective effects of BIS. The i.t. instillation mode of delivery of nanoparticles has been shown to be valid and appropriate as it allows precise dosing of particles and is considered suitable for exploring the action mechanisms of the particles (Morimoto et al., 2016, 2018). The dose of DEPs used in the present study was based on our previous work and is similar to the particulate air pollutant doses used in prior experimental animal studies (Nemmar et al., 2015; Mutlu et al., 2007; Kido et al., 2011).

Human and animal experimental studies have substantiated the occurrence of airway hyperresponsiveness and inflammation following exposure to DEPs (Nemmar et al., 2018; Ghio et al., 2012; Kim et al., 2016; Kayalar et al., 2024). In the present study, we confirm the manifestation of airway hyperresponsiveness and show for the first time that treatment with BIS (at both 25 and 50 mg/kg) markedly resulted in its prevention upon exposure to DEPs through i.t. instillation. By using rat tracheal rings pretreated with BIS and challenged with ovalbumin, it was demonstrated that BIS mitigated the hyperresponsiveness through inhibition of the voltage-dependent Ca^2+^ channels (de Siqueira et al., 2012). Moreover, it has been reported that treatment with nanoencapsulated BIS (30–100 mg/kg) significantly reduces lipopolysaccharide (LPS)-induced airway hyperreactivity and pulmonary neutrophilic inflammation (D'Almeida et al., 2017). In the present study, acute exposure to DEPs induced significant influxes of macrophages, neutrophils, and lymphocytes as well as elevated Evans Blue extravasation and total proteins in the BALF besides causing airway hyperresponsiveness, indicating the increase in alveolar–capillary barrier permeability. The latter effects explain the infiltration of inflammatory cells (macrophages, neutrophils, and lymphocytes) in the BALF into the alveolar space through the damaged endothelium and epithelium. These pulmonary pathophysiological effects were mitigated with BIS treatment at both doses. Our data corroborate the results of a recent study that showed that BIS reduces leukocyte recruitment in the peritoneal cavity of the caecal-ligation- and puncture-induced systemic infection model in mice (Cavalcante et al., 2020). It is well-established that the recruitment and activation of inflammatory cells in the lungs can cause overproduction of proinflammatory cytokines, reactive oxygen species (ROS), and the granular enzyme MPO, all of which play essential roles in prompting and promoting pulmonary inflammation (Hoenderdos and Condliffe, 2013; Addissouky et al., 2024). Herein, we found that acute exposure to DEPs induced significant increases in the production of proinflammatory cytokines (TNFα, IL-1β, and IL-6) as well as the levels of the marker LPO, antioxidants SOD and GSH, and free-radical scavenger NO. Interestingly, pretreatment with BIS significantly prevented pulmonary inflammation as well as oxidative and nitrosative stresses, confirming the effective antioxidant and anti-inflammatory actions of BIS. It was also shown that BIS exerts protective effects against cisplatin-induced nephrotoxicity in mice, β-adrenergic agonist-induced myocardial infarction in rats, and LPS-induced lung injury in mice through mechanisms involving inflammation and oxidative stress (Zaaba et al., 2022; Meeran et al., 2020; D'Almeida et al., 2017).

Metabolic alterations due to amplified mitochondrial dysfunctions under inflammatory and oxidative stress conditions have been increasingly recognized in various pathophysiological conditions (Bennett et al., 2022; Pokharel et al., 2024). In this context, consistent associations have been reported between mitochondrial dysfunction and lung injuries, such as inflammatory cell recruitment, proinflammatory cytokine release, and increased alveolar capillary permeability (Long et al., 2022). Our data show that pulmonary exposure to DEPs significantly increased the levels of mitochondrial respiratory complexes I, II, III, and IV. It has been reported that exposure of the human bronchial epithelial cell line BEAS-2B to cigarette smoke extracts causes structural and functional alterations in the mitochondria, including elevation of the oxidative phosphorylation proteins (complexes II, III, and V), oxidative stress (Mn-SOD) markers, and concentrations of proinflammatory cytokines (IL-6, IL-1β, and IL-8) (Hoffmann et al., 2013). Furthermore, cigarette smoke exposure in mice has been shown to augment the activities of complexes II, III, and IV as well as ATPase in the lungs (Agarwal et al., 2012). Interestingly, our findings show that administration of the antioxidant and anti-inflammatory BIS significantly alleviated DEP-induced mitochondrial dysfunction in the lung. BIS has been reported to abrogate isoproterenol-induced myocardial mitochondrial dysfunction (Meeran et al., 2019).

Various studies have established that exposure to particulate air pollution can induce genotoxicity both *in vivo* and *in vitro*. It is well-documented that various toxic particulate matter constituents (e.g., transition metals and polycyclic aromatic hydrocarbons) can generate ROS that can cause oxidative stress at the cellular level, induce bulky DNA adducts, and oxidative DNA damage (Moller et al., 2014; Quezada-Maldonado et al., 2021). Our data show that acute exposure to DEPs induces DNA damage, which can be linked to the observed inflammation and oxidative stress actions. Hence, the use of the anti-inflammatory and antioxidant BIS potently ameliorates such effects. BIS has been reported to exert antioxidant and antigenotoxic effects *in vitro* and protect against DNA damage in a mouse model of cisplatin-induced nephrotoxicity *in vivo* (Zaaba et al., 2022; Anter et al., 2011). Following the induction of DNA damage, a prominent route of cell inactivation is apoptosis. An important physiological function of apoptosis is to remove injured cells that have endured DNA damage. The breakdown of these cell structures is achieved by a series of cysteine proteases called caspases, particularly caspase-3, and other enzymes (Pandey et al., 2017; Savitskaya and Onishchenko, 2015). Active caspase-3 is possibly implicated in most alveolar apoptotic processes, and the assessment of caspase-3 is instrumental in the documentation of apoptosis at the tissue and cellular levels (Pandey et al., 2017; Savitskaya and Onishchenko, 2015). Our data show that DEPs significantly increased the level of cleaved caspase-3 and that BIS pretreatment significantly reduced this effect. It has been reported that nootkatone, a constituent of grapefruit that has anti-inflammatory and antioxidant actions, prevents caspase-3 activation induced by DEPs while inhibiting pulmonary inflammation and oxidative damage (Nemmar et al., 2018).

To better understand the mechanisms by which BIS alleviates these effects, we assessed JNK expression by Western blot. JNK is a stress-activated protein kinase that is stimulated by environmental causes, such as ROS, inflammatory cytokines, and growth factors (Bennett, 2006). In fact, JNK has been reported to be involved in various lung diseases, including pollutant-induced bronchitis, allergic and non-allergic asthma, chronic obstructive pulmonary disease, and fibrosis (Bennett, 2006). An interesting finding of the present work was that the JNK signaling pathway was noticeably activated following exposure to DEPs. Remarkably, pretreatment with BIS reduced the expression of JNK, suggesting that this pathway plays an important role in the protective effects of BIS. The production of proinflammatory cytokines and ROS has been shown to trigger the JNK pathway; hence, we speculate that inflammation and oxidative stress could be the upstream mediators of the JNK signaling pathway (Rahman and MacNee, 2000). Our data corroborates the findings of a recent study that showed that elevated phosphorylation of JNK in ovarian cancer was significantly reversed by pretreatment with N-acetylcysteine (Zhang et al., 2022). Moreover, it has been reported that JNK inhibition plays an important role in the protective effects of taurine, an intracellular free β-amino acid, against doxorubicin-induced cardiac oxidative stress and apoptosis (Das et al., 2011).

The current study has certain limitations. We evaluated the protective effects of BIS following an acute single-dose exposure to DEPs but it would be valuable to extend this research to examine its impacts under chronic exposure to repeated DEP doses. Additionally, exploring whether BIS mitigates the extrapulmonary effects resulting from pulmonary DEP exposure can provide deeper insights into the protective effects of BIS. Future studies can also validate our findings in animal models of other respiratory diseases, such as asthma, chronic obstructive pulmonary disease, pulmonary hypertension, and pulmonary fibrosis.

## Conclusion

In summary, our findings demonstrate that BIS markedly ameliorates DEP-induced lung injury by regulating inflammatory, oxidative stress, and apoptotic biomarkers through the JNK signaling pathway. Pursuant to additional studies, BIS can be considered as a plausible protective agent against inhaled-particle-induced pulmonary adverse effects.

## Data Availability

The original contributions presented in the study are included in the article/supplementary material, further inquiries can be directed to the corresponding author.
